# Bonding strength and fatigue survival of conventional, additive and subtractive complete dentures

**DOI:** 10.1038/s41598-026-44101-7

**Published:** 2026-03-18

**Authors:** Jörg Lüchtenborg, Andreas Keßler, Florian Schneider, Gregor Kleinvogel, Anna-Lena Hillebrecht, Kirstin Vach, Benedikt C. Spies

**Affiliations:** 1 Department of Prosthetic Dentistry, Center for Dental Medicine, Faculty of Medicine, Medical Center - University of Freiburg, University of Freiburg, Hugstetterstr. 55, 79106 Freiburg, Germany; 2 Institute of Medical Biometry and Statistics, Faculty of Medicine, University of Freiburg, Stefan-Meier Str. 26, 79104 Freiburg, Germany; 3 Private Practice, Clara-Zetkin-Straße 16, Beelitz, Germany

**Keywords:** Denture base, Denture teeth, CAD/CAM, Bond strength, Thermomechanical aging, Shear fracture, 3D printing, PMMA, Subtractive manufacturing, Engineering, Health care, Materials science, Medical research

## Abstract

This study evaluated the bonding strength, fatigue survival, and fracture modes of dentures fabricated through conventional, additive (SLA/DLP), and subtractive CAD/CAM workflows to test their clinical durability after simulated oral ageing. Eight denture base-tooth combinations (*n* = 8 per group) were produced using either heat-polymerised PMMA, additively manufactured resins (Formlabs Denture Base LP or Voco V-Print Dentbase) or subtractively milled PMMA (Voco CediTec DB). Prefabricated (VITA Vionic) or CAD/CAM-fabricated denture teeth were then bonded. Surface roughness was quantified using laser scanning microscopy and the degree of conversion using Raman spectroscopy. The specimens underwent hydrothermal ageing with dynamic loading (1.2 million cycles at 5 °C/55 °C) followed by quasi-static shear fracture testing and fracture mode analysis. The conventional fabricated cast-on samples achieved the highest mean fracture load both before (445.7 ± 49.2 N) and after aging (411.7 ± 45.7 N) with 100% survival. Additive workflows (SLA/DLP) groups showed cohesive failures pre-aging; however, the DLP workflow demonstrated a 57% strength reduction post-aging (*p* = 0.0057) and 87.5% survival. Subtractive groups had the lowest survival rates (50–62.5%), significant strength losses (*p* < 0.05), and predominantly adhesive failures at the tooth-base interface. Ageing significantly affected bond strength in selected additive and subtractive groups (*p* < 0.05), whereas the conventional cast-on group remained unaffected. Conventional heat-polymerized PMMA bonded to prefabricated teeth remains the benchmark for denture fabrication. Additive CAD/CAM workflows approach similar performance, whereas subtractive methods require further optimization to improve bond stability under hydrothermal fatigue.

## Introduction

The integrity of the bond between denture base materials and artificial teeth is a critical determinant of complete denture longevity, functional performance and patient satisfaction. Tooth debonding remains among the common causes of denture repair, with earlier studies reporting complication rates of approximately 30%^1^. Such failures not only compromise function and esthetics but also increase maintenance costs and the treatment burden, particularly for older adults who rely to a significant degree on removable prostheses. Recent advancements in digital denture fabrication technologies have transformed clinical workflows by improving reproducibility, reducing chair time and standardizing manufacturing processes. Computer-aided design and computer-aided manufacturing (CAD/CAM) systems allow denture bases to be produced either subtractively from pre-polymerized PMMA blocks or additively using layer-based technologies such as stereolithography (SLA) and digital light processing (DLP)^2,3^. While subtractive workflows provide homogeneous material structures due to industrial pre-polymerization, additive manufacturing consists of layerwise photopolymerization where the mechanical behavior may be influenced by interlayer adhesion and post-curing conditions. Considerable research has focused on improving the intrinsic mechanical properties of denture base polymers. Strategies include the incorporation of nanofillers or nanotubes to enhance impact strength, hardness and antimicrobial properties, as well as the development of polymeric interpenetrating networks such as polyurethane-modified systems to improve flexural strength and fatigue resistance^4,5^.

Although such approaches enhance bulk mechanical performance, they do not necessarily address the integrity of adhesive interfaces. In conventional heat-polymerized dentures, denture teeth are integrated during polymerization, allowing chemical interpenetration between materials and potentially cohesive bonding at the interface. In contrast, CAD/CAM fabrication typically involves separate production of denture bases and teeth followed by adhesive bonding between pre-polymerized components^3^. This procedural difference shifts the critical region of mechanical performance from the bulk material to the adhesive interface. Studies on additively manufactured polymer structures have demonstrated that structural failure often initiates within bonded overlap regions due to interfacial stresses and peel forces. In such systems, structural integrity is governed primarily by the quality of the adhesive interface rather than by bulk mechanical properties^6^. These findings suggest that, particularly for layer-based manufacturing techniques, optimization of the tooth–base interface may be decisive for long-term durability. Although CAD/CAM-fabricated dentures have demonstrated improved fit and promising mechanical properties^2,7^, the bonding performance between CAD/CAM fabricated denture bases and artificial teeth, which can be measured through static adhesion tests conducted in accordance with DIN ISO/TS 19736, is reported differently in various studies. Some investigations demonstrated comparable shear bond strength to conventional systems^3^, whereas others reported reduced bond strength following thermomechanical ageing^8^. Moreover, many available studies focus on static bond strength testing without combining fracture load evaluation with ageing. Therefore, testing protocols that integrate static fracture testing with thermomechanical fatigue simulation are required to approximate intraoral loading conditions and hydrothermal degradation.

The objective of this study was to compare the bonding strength, fatigue survival and fracture modes of dentures fabricated using conventional heat-polymerized PMMA, additive CAD/CAM workflows (SLA and DLP), and subtractive CAD/CAM workflows, in combination with prefabricated and CAD/CAM-manufactured denture teeth following hydrothermal ageing and dynamic loading to simulate the oral environment.

The null hypotheses is therefore, that (1) there is no difference in bond strength between prefabricated denture teeth bonded to conventionally fabricated denture bases and those bonded to digitally fabricated denture bases and (2) there is no difference in adhesive bonding performance between CAD/CAM-manufactured dentures (additive or subtractive) and conventionally fabricated dentures.

## Materials and methods

**Fig. 1:**
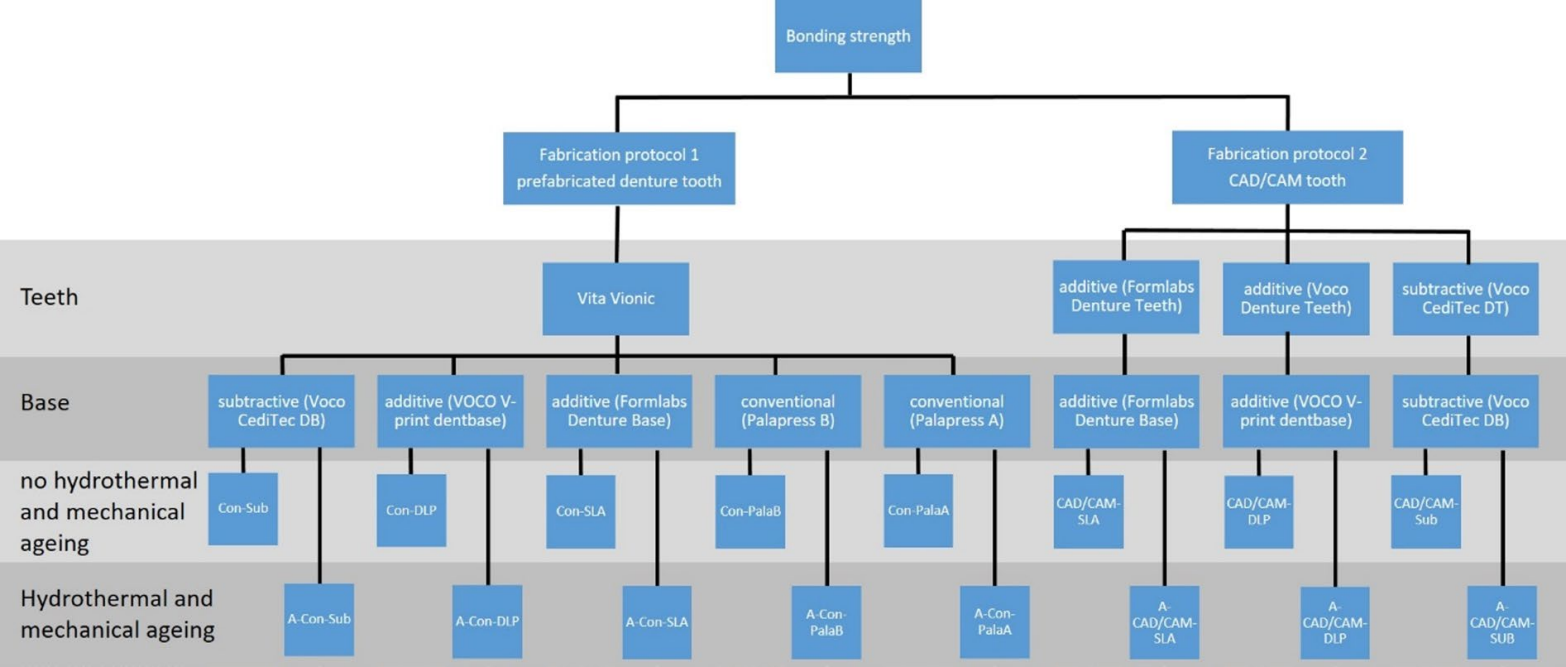
Experimental setup of the investigation.

The experimental setup of the study is depicted in Fig. 1.

### Sample preparation

The Vita Vionic denture tooth (Vita, Bad Säckingen, Germany), scanned and subsequently designed using the Fusion360 program (Autodesk, San Francisco, USA), was subtracted from a cylindrical base shape (height: 20 mm, diameter: 25 mm) using Boolean difference to generate an STL file of the base.

The test specimens of the base were produced from a conventional denture base material (PalaXpress, Kulzer, Hanau, Germany), two additively manufactured materials (Voco V-Print Dentbase, Voco, Cuxhaven, Germany and Formlabs Denture Base, Formlabs, Sommerville, USA) and a subtractively manufactured material (Voco CediTec DB, Voco, Cuxhaven, Germany), with *n* = 8.

An STL file of the corresponding mould was then printed and duplicated for the conventional group. In order to test the Pala A group, the cast-on test specimen base with tooth was duplicated. The tooth was then embedded in a hollow mould and cast on PalaXpress. In the case of the Pala B group, the base was duplicated, moulded with PalaXpress and the Vita tooth was bonded with CediTec Adhesive. The curing process was conducted for 30 min in a pressure pot maintained at 55 °C and 2 bar. For the subtractive group, the STL file was nested in inLab and fabricated from the material CediTEC DB using the 5-axis machine MCX5 (Dentsply Sirona, Bensheim, Germany). The support structures were then removed and ground smooth.

The test specimens were manufactured using stereolithography (SLA) and digital light processing (DLP) 3D-printing processes, with the CAM software Preform or Netfabb employed to create the printing data. The support structures and printing parameters were selected (layer thickness 50 μm) and the specimens were manufactured for the materials Formlabs Denture Base LP and Voco Dentbase, respectively. The Formlabs Denture Base LP specimens were manufactured using the Form 3 SLA printer (Formlabs, Sommerville, USA), while the Voco Dentbase specimens were manufactured using the DLP printer D20II (Rapidshape, Heimsheim, Germany). Subsequently, the specimens were subjected to post-processing in accordance with the instructions provided by the manufacturer. The support structures were then removed.

The prefabricated VITA VIONIC teeth were then bonded into the corresponding test cylinders of the denture base using Voco CediTec Adhesive.

In the second part of the study, the denture bases were bonded to CAD/CAM meaning subtractively or additively manufactured denture teeth. In the subtractive group, the STL file of the teeth and bases was generated in inLab and fabricated using a 5-axis machine, MCX5 (Dentsply Sirona, Bensheim, Germany), from the materials CediTEC DT and CediTEC DB (Voco, Cuxhaven, Germany). The support structures were then removed and ground until a smooth surface was achieved. The test specimens intended for additive manufacturing were constructed using Preform or Netfabb, with the corresponding support structures and printing parameters selected (layer thickness 50 μm) and employed for the material Formlabs Denture Teeth and Denture Base using a SLA printer (Form 3, Formlabs, Sommerville, USA). The specimens with the material Voco Denture Teeth and the base material Voco V-print Dentbase were printed using a DLP printer (D20II, Rapidshape, Heimsheim, Germany). The subsequent post-processing was carried out in accordance with the instructions provided by the manufacturers.

### Post-processing base material

The samples of Formlabs Denture Base LP material were removed from the build platform and cleaned in isopropanol for a period of 10 min (Form Wash, 99.9% Höfer Chemie, Kleinblittersdorf, Germany). Following drying, the support structures were removed and washed for a further five minutes. Subsequently, the tooth tray was moistened with denture base resin, and the Vita Vionic tooth was transferred using a fixation template. While the tooth was affixed within the tooth socket using the aforementioned fixation template, the contact area was cured using a portable UV light-curing unit (Elipar Deep cure, 3 M ESPE, Kamen, Germany). Subsequently, additional post-curing was conducted in a glycerine bath (Form Cure, 80 °C, 2 × 30 min).

The Voco V-print dentbase material was subjected to post-curing in the following manner: Cleaning was conducted using isopropanol (99.9% Höfer Chemie GmbH) in the RS Wash (Rapidshape, Heimsheim, Germany) apparatus, with a 3-minute pre-wash and subsequent 2-minute cleaning in fresh isopropanol. Subsequently, the support structures were meticulously eliminated, and following a 15-minute interval to permit the residual isopropanol to dissipate, post-exposure was conducted for 30 min (RSCure, Rapidshape, Heimsheim, Germany).

### Preparation of the bonding surface

To prepare the bonding surface of the Vita Vionic teeth with the model base, the bonding surface of the base and the bonding surface of the CAD/CAM-fabricated denture teeth were roughened with aluminium oxide (2 bar / 50 μm). The Vionic teeth were bonded with the aid of CediTEC Primer & Adhesive and a fixation template.

The denture base and teeth were each blasted with aluminium oxide (particle size: 50 μm) at 2 bar before bonding and then evaporated. The subtractively fabricated CediTec DT teeth and the CediTec DB base were coated with CediTec Primer according to the manufacturer’s instructions, air-dried for 30 s and then bonded using CediTEC Adhesive. The specimens were then placed in a pressure pot at 2 bar and 55 °C for 15 min using a composite mould that generated a 2 kg load. The printed teeth made from the Voco denture teeth material and the Voco V-print Dentbase printed base were produced simultaneously.

The Formlabs Denture Teeth additively manufactured in mould were bonded directly to the Denture Base material using the Denture Base resin. While the tooth was fixed in the tooth tray using the fixation template, the area where the tooth and base touch was cured using a portable UV light curing unit (with a measured output of 1280 mW/cm^2^). Finally, post-curing was carried out in the Form Cure for 60 min at 80 °C in Glycerin.

### Roughness measurement

To determine the roughness of the composite surface, the surfaces of the different bases were blasted (2 bar, 50 μm Al2O3) and characterised using a laser scanning microscope (VHX 3030, Keyence GmbH, Neu-Isenburg, Germany). The materials Voco V-print dentbase (V-Base) and Formlabs Denture Base (F-Base) were printed horizontally with 0° and at an angle of 45° to the build platform. PalaXpress (Pala) was moulded for the conventionally produced specimens in the same way as for the composite specimens. For this purpose, a printed plate with a print orientation of 0° and 45° was duplicated and moulded with PalaXpress. For the samples of the subtractive production group, a surface was milled out of the blank Voco CediTec DB (V-Sub).

### Degree of conversion

The Degree of conversion was determined using Raman measurement (inVia Qontor, Renishaw plc, New Mills, England). The measurements were carried out using a laser with a wavelength of 785 nm at 100% power and 10 s integration time with 3 repetitions in each case. Five spectra each of the resin and the post-cured samples were recorded in the range between 1100 and 2000 cm^− 1^ and the ratio of the unpolymerized methacrylate double bond at 1637 cm^− 1^ to the aromatic C = C double bond at 1609 cm^− 1^ was used. The degree of conversion results from the following formula:

$$\mathrm{D}\mathrm{C}\left[{\%}\right]=100\mathrm{*}\left(1-\frac{{R}_{cured}}{{R}_{Resin}}\right);\quad R=\frac{{I}_{1637}}{{I}_{1609}}$$

### Hydrothermal ageing and dynamic loading

**Fig. 2:**
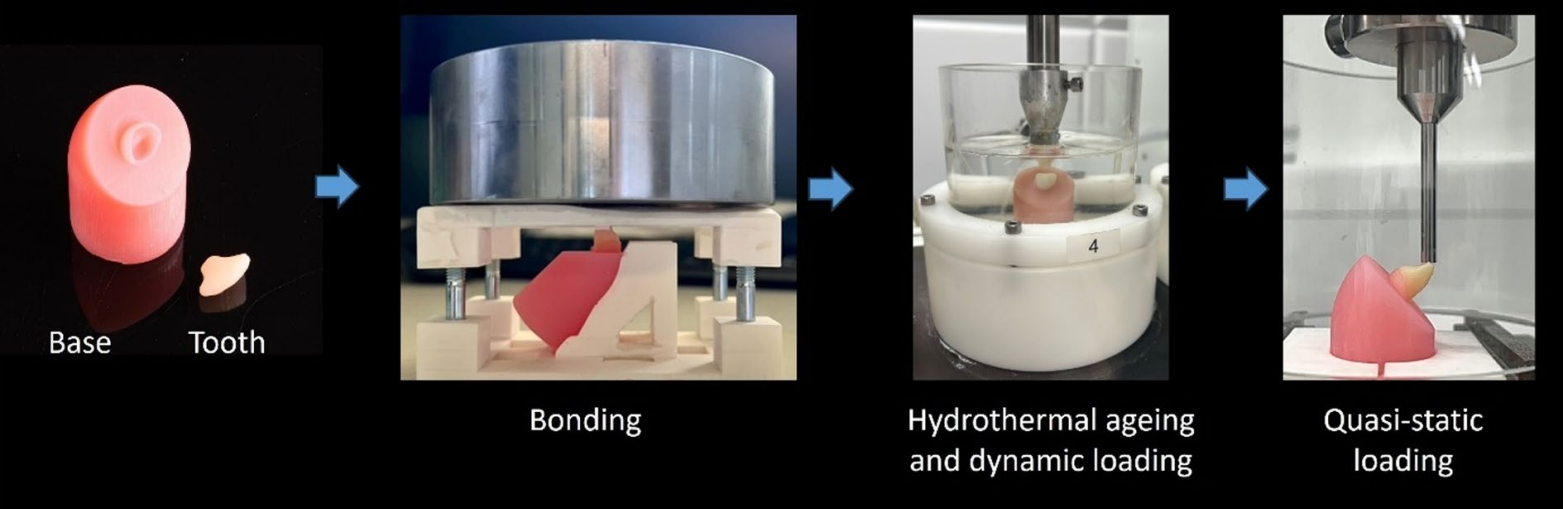
Images of the manufactured tooth and base, the setup for bonding with a defined load of 2 kg, the setup during hydrothermal ageing and dynamic loading and the testing setup for quasi-static loading with a shear angle of 135°.

Four samples from each of the eight groups were aged hydrothermally (5 °C (30s)/55 °C (30 s)) while subjected to dynamic loading for 1.2 million cycles with a force of 49 N at a frequency of 1.3 Hz in a chewing simulator (CS-4.8 professional line, SD-Mechatronik, Feldkirchen-Westerham, Germany) (Fig. 2).

### Bonding strength

**Fig. 3:**
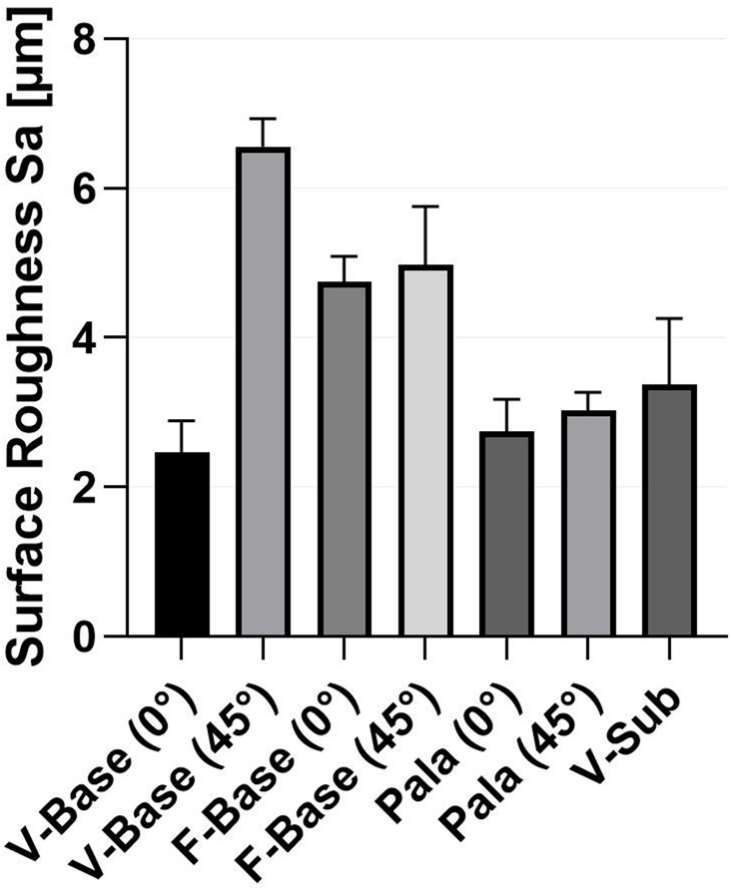
Roughness of the surface of the sandblasted base materials.

To determine the maximum fracture load, the specimens were quasi-statically loaded to fracture using a universal testing machine (Z010, ZwickRoell GmbH, Ulm, Germany). The testing device according to ISO 19736 is shown in Fig. 3. The force was applied under displacement-controlled conditions at a speed of 10 mm/min until the specimen fractured. The shear angle was equal to the interinsic angle of 135°. During the test, the force (in N) and displacement (in mm) of the frame were recorded over time. The examination and photographic documentation of the fracture surfaces was carried out under 20x magnification (VHX 7000 N, Keyence GmbH, Neu-Isenburg, Germany). The fracture types were evaluated and classified as follows: (1) Adhesive failure (AF), when the fracture clearly occurred along the interface between the tooth and the base; (2) Cohesive failure (CF), when tooth remnants remained bonded to the base resin or resin remnants remained bonded to the tooth; (3) Mixed (MF), when significant areas of adhesive and cohesive failure occurred simultaneously.

### Statistical analyses

A priori sample size calculation was performed based on data obtained from a pilot (pre-test) experiment. Because no reliable effect size estimates for the investigated material combinations were available in the literature, pilot data were used to estimate variability and the expected difference in bond strength between groups. The calculation was based on detecting a minimum difference of 100 N in fracture load at a significance level of α = 0.05 and a statistical power of 80%, resulting in a required sample size of six specimens per group. To compensate for potential specimen loss during ageing, eight specimens per group were included. For descriptive analysis median, mean values and standard deviations were calculated. Kaplan-Meier survival rates were computed for the number of cycles done until fracture. Due to a deviation from the normal distribution Dunn tests with Holm correction were used to compare the bonding strength between groups. To evaluate the influence of aging or fabrication protocol in subgroups Wilcoxon rank-sum test was applied.

The data were analyzed using STATA (version 17.0, College Station, TX, USA) with a significance level of 5%.

## Results

### Roughness of the base-materials

The roughness values Sa for the different base materials are shown in Fig. 3. The 45° orientation shows higher surface roughness than the 0° orientation, with the Sa between 2.5 ± 0.3 μm (V-Base 0°) to 6.6 μm ± 0.3 (V-Base 45°).

### Degree of conversion

**Fig. 4:**
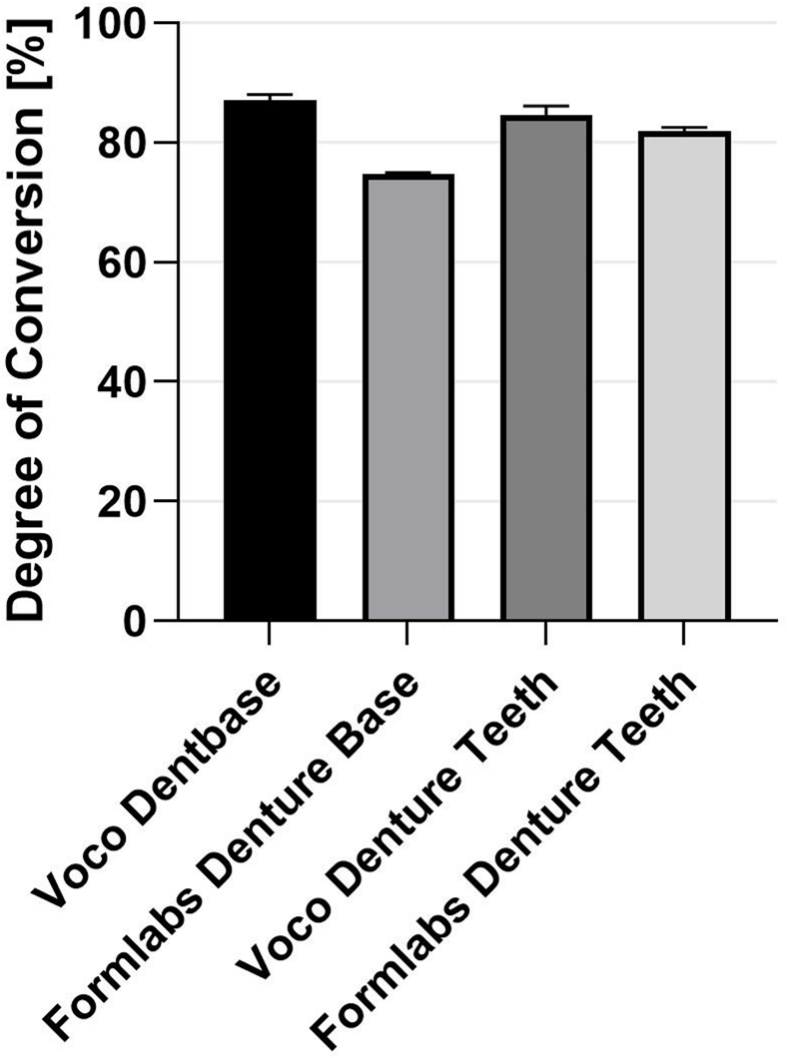
Degree of conversion of the printed materials measured with Raman microscopy.

The Degree of conversion of the bonded surface of the printed samples determined by Raman microscopy is shown in Fig. 4 and Table 1. The printed surfaces with the material Voco Dentbase and Voco Denture Teeth show a higher conversion of the double bond of 87,4 ± 0,4% and 85,3 ± 0,6%compared to the printed samples with the material Formlabs Denture Base (74,7 ± 0,2%) and Formlabs Denture Teeth (82,2 ± 0,3%).

**Table 1:** Degree of conversion of the printed materials.

	Voco dentbase	Formlabs denture base	Voco denture teeth	Formlabs denture teeth
Degree of conversion [%]	87,4 ± 0,4	74,7 ± 0,2	85,3 ± 0,6	82,2 ± 0,3

### Hydrothermal ageing and dynamic loading

**Fig. 5:**
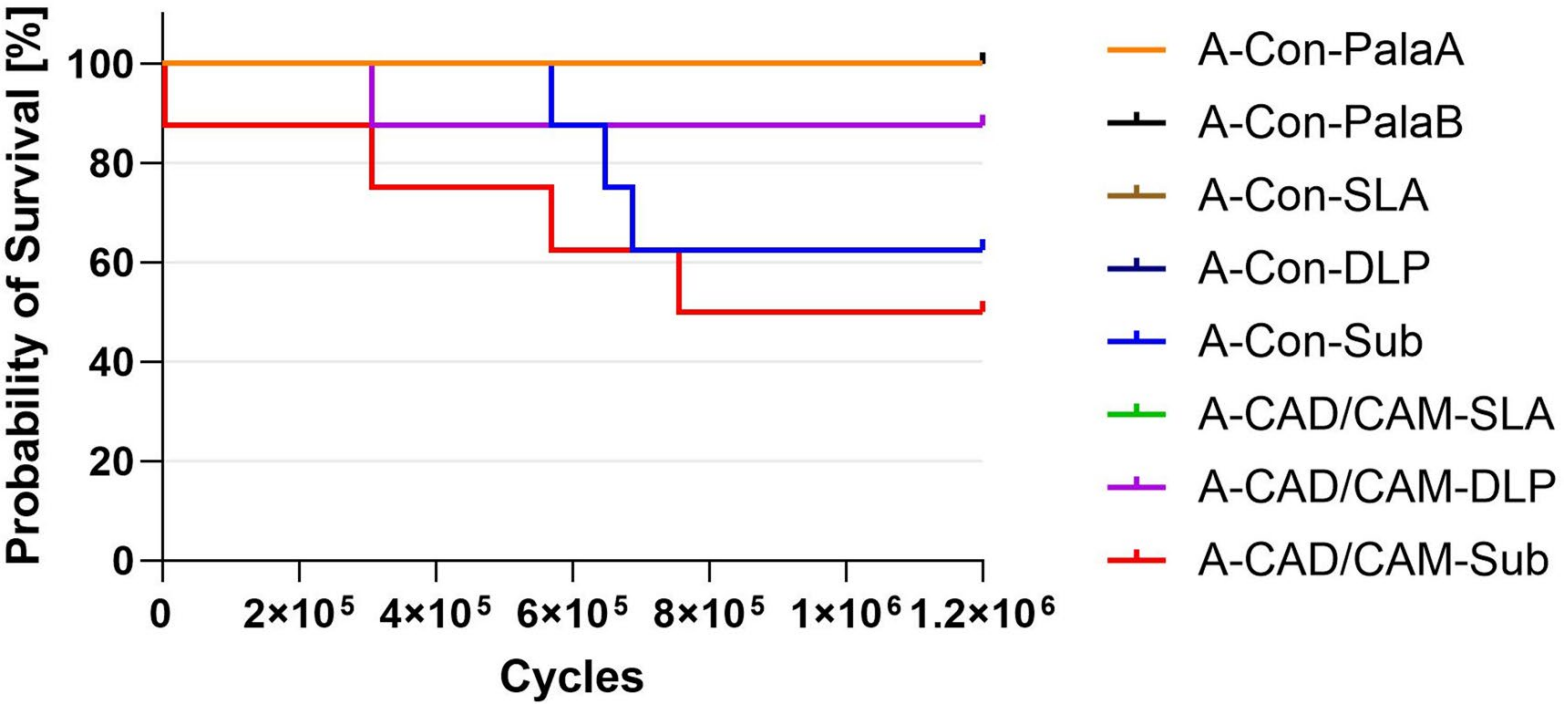
Kaplan Meier Plot for the survival probability after hydrothermal ageing and dynamic loading.

The probability of survival during hydrothermal ageing and dynamic loading is shown graphically in a Kaplan-Meier diagram in Fig. 5.

The survival probability of the A-CAD/CAM-DLP group after 1.2 million cycles under dynamic loading in hydrothermal environment is 87.5%. The A-Con-Sub group exhibits a survival probability of 62.5% after 1.2 million cycles. The samples of the A-CAD/CAM-Sub group demonstrate a survival probability of 50% after 1.2 million cycles. In contrast, the survival probability for all other groups is 100% after the same number of cycles.

### Bonding strength

**Fig. 6:**
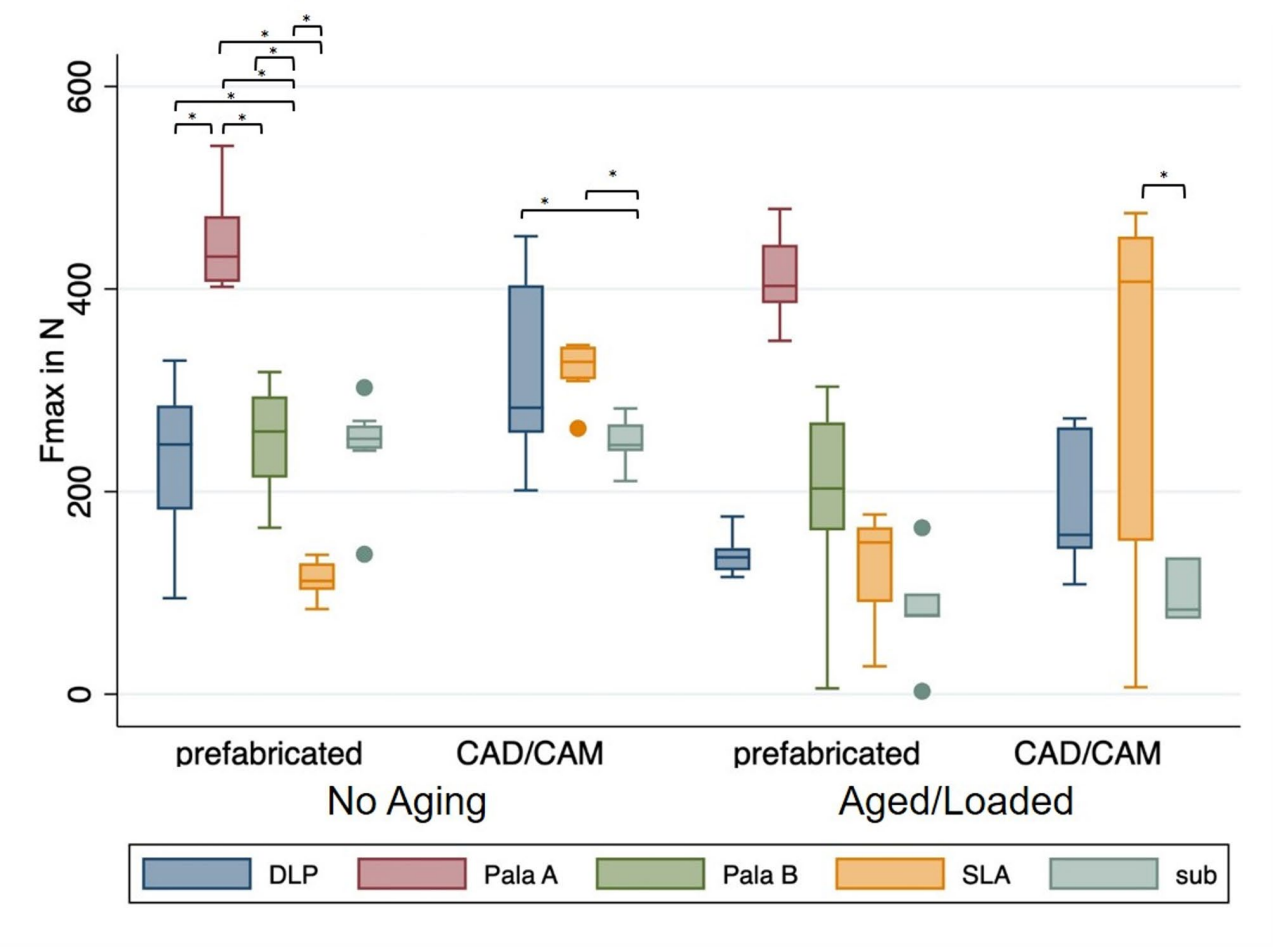
Bonding strength for the different groups.

**Fig. 7:**
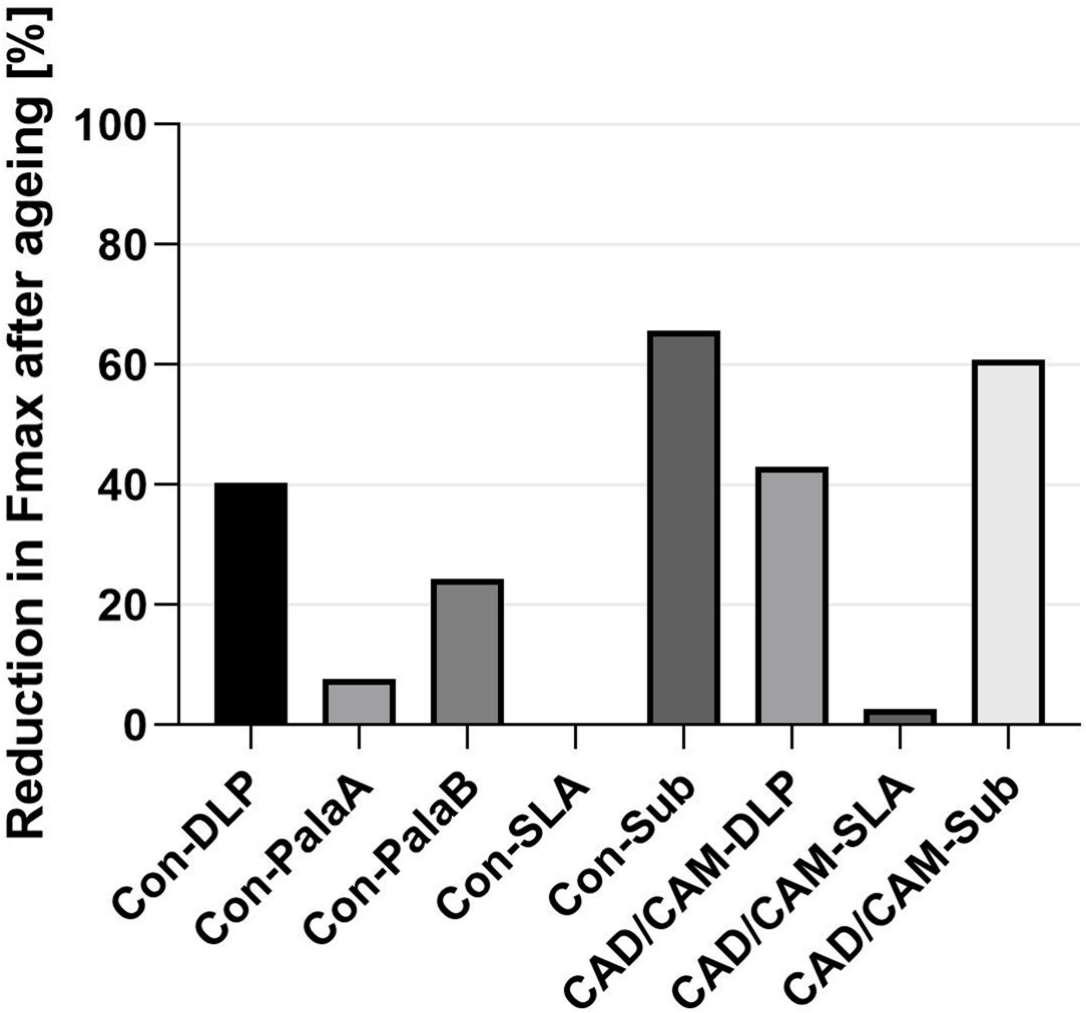
Percentage decrease in Fmax in comparison before and after ageing.

The bonding strength of the different groups is shown in Fig. 6. and the reduction of the bonding strength in presented in Fig 7. 

### Prefabricated denture tooth

#### Without hydrothermal ageing and dynamic loading

In the groups with prefabricated teeth, the specimens in the Con-PalaA group achieved the highest bond strength of 445.7 ± 49.2 N, which differs significantly from the Con-DLP group with 231.5 ± 76.8 N (*p* = 0.0108). At 252.1 ± 52.9 N, the Con-PalaB group shows a significant difference to Con-PalaA. The Con-SLA group with 113.7 ± 18.0 N is significantly different from the Con-DLP group with 231.5 ± 76.8 N (*p* = 0.0443); Con-PalaA with 445.7 ± 49.2 N (*p* = 0.0) and Con-PalaB with 252.1 ± 52.9 N (*p* = 0.0203). The Con-Sub group shows a bond strength of 245.0 ± 47.0 N and differs significantly from the Con-PalaA (*p* = 0.0232) and Con-SLA (*p* = 0.0257) groups.

#### With hydrothermal ageing and dynamic loading

After hydrothermal ageing and dynamic loading in the chewing simulator, the A-Con-PalaA group with a bond strength of 411.7 ± 45.7 N shows a significant difference to the A-Con-DLP group with 137.9 ± 21.4 N (*p* = 0.0057). The A-Con-SLA group (122.1 ± 62.4 N) differs significantly from the A-Con-PalaA group (*p* = 0.0092). The A-Con-Sub group (84.1 ± 57.0 N) also differed significantly from the A-Con-PalaA group (*p* = 0.0003).

### CAD/CAM-fabricated denture tooth

#### Without hydrothermal ageing and dynamic loading

The results of the CAD/CAM-Sub group show a significant difference compared to the CAD/CAM-DLP (317 ± 90.0 N; *p* = 0.0403) and CAD/CAM-SLA (321.4 ± 28 N; *p* = 0.0070) groups.

#### With hydrothermal ageing and dynamic loading

The A-CAD/CAM-Sub group shows a significant difference (*p* = 0.0225) in bond strength after the chewing machine with 97.7 ± 32.7 N compared to the A-CAD/CAM-SLA group with 312.8 ± 180.8 N.

### Influence of hydrothermal ageing and dynamic loading on the bonding strength

The evaluation of the groups with the prefabricated Vita-Vionic denture tooth, showed that the Con-DLP (*p* = 0.0293) and Con-Sub (*p* = 0.0031) groups exhibited a significant difference in the maximum fracture force between the specimens not hydrothermal aged and dynamic loaded and the specimens which underwent hydrothermal ageing and dynamic laoding. The Con-PalaA, Con-PalaB and Con-SLA groups showed no significant difference between aged and non-aged samples.

With the CAD/CAM-fabricated teeth, the CAD/CAM-DLP (*p* = 0.093) and CAD/CAM-Sub (*p* = 0.0121) groups showed a significant difference in the maximum fracture force between the samples with and without hydrothermal ageing and dynamic loading.

### Influence of the fabrication protocol

In the SLA group (*p* = 0.0002), there was a significant difference in bond strength between groups with prefabricated tooth and groups with a CAD/CAM-fabricated denture tooth, before ageing. After hydrothermal ageing and dynamic loading, no significant difference in bond strength was found between the groups with a prefabricated and CAD/CAM-fabricated tooth.

### Fractography

**Fig. 8:**
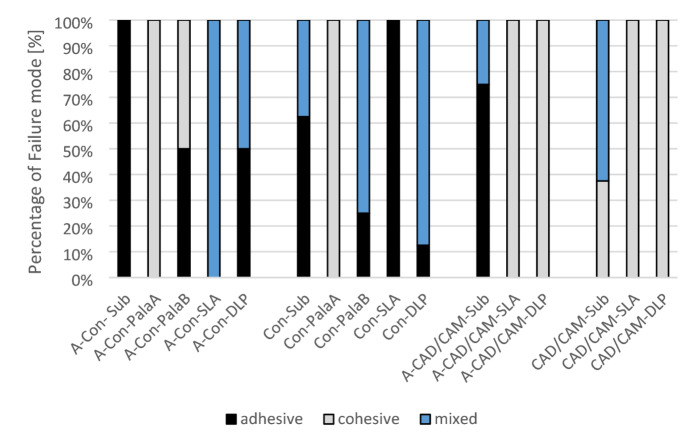
Proportion of adhesive fracture, cohesive fracture and mixed fracture for the different groups.

**Fig. 9:**
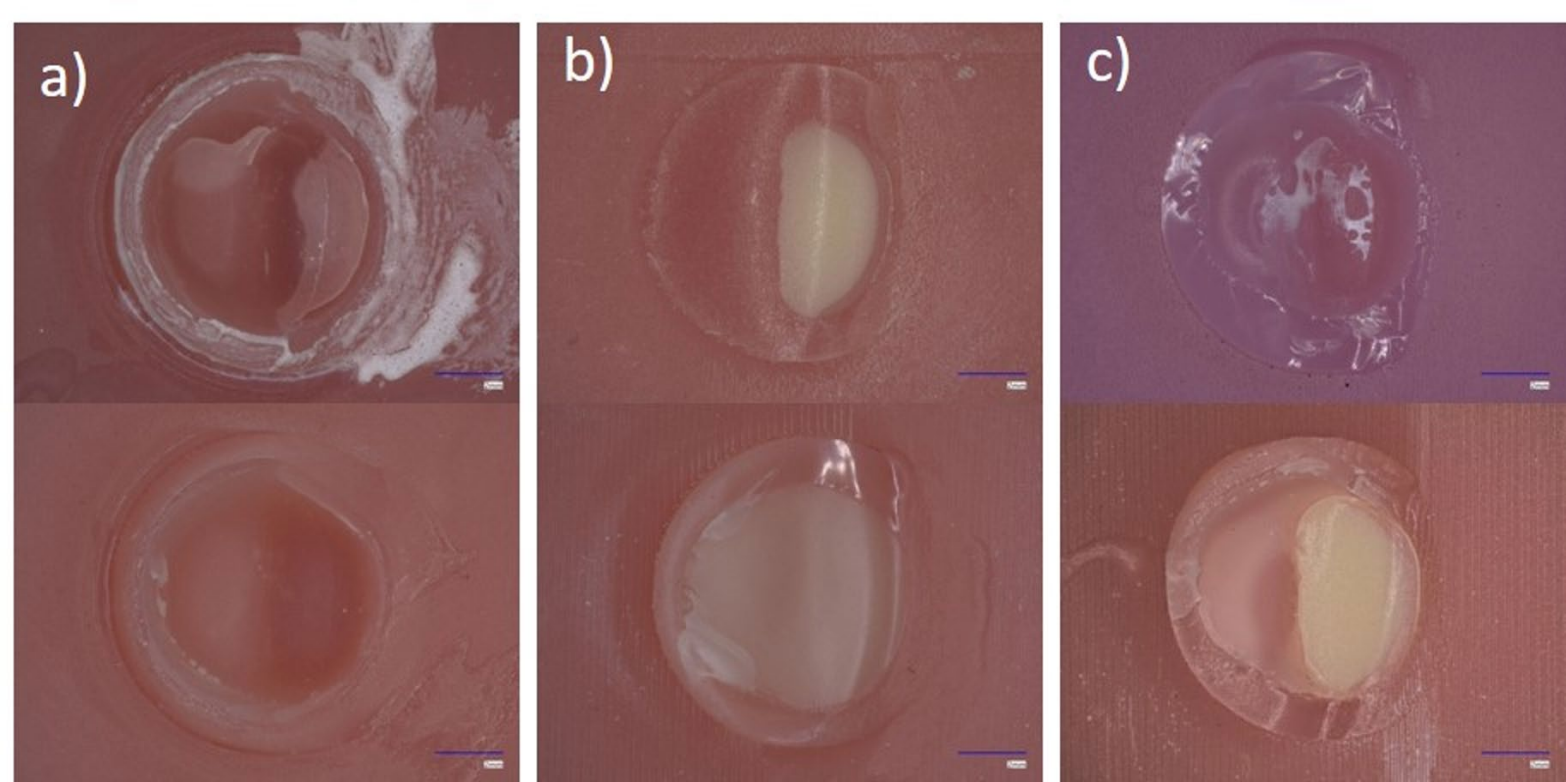
Exemplary representation of the fracture types (**a**) adhesive, (**b**) cohesive, (**c**) mixed.

The different fracture types for each group are shown in Table 2; Fig. 8. Exemplary samples for the respective failure type are shown in Fig. 9. The fracture of the samples with the base manufactured by SLA showed 100% adhesive fracture (Con-SLA) which changed after ageing (A-Con-SLA) to 100% mixed fracture. For DLP an increase from 12.5% adhesive fracture (Con-DLP) to 50% adhesive fracture (A-Con-DLP) after ageing could be observed. Subtractive groups showed in with the prefabricated tooth mostly adhesive failure (without ageing 62,5% and after ageing 100%). Con-Pala-A showed a cohesive fracture nevertheless it underwent ageing or not. Con-Pala-B from 75% cohesive and 25% adhesive fracture to 50% adhesive and 50% cohesive fracture pattern after hydrothermal ageing and dynamic loading. The group CAD/CAM-Sub changed from 62,5% mixed fracture to 75% adhesive for the sample subjected to hydrothermal ageing and dynamic loading. Neither of the CAD/CAM-SLA nor the CAD/CAM-DLP groups changed the fracture mode, which remained at 100% cohesive.

**Table 2:** Fracture mode of the different groups.

Group	Treatment	Teeth	Base	Fracture mode					
				Adhesive		Cohesive		Mixed	
				n	%	n	%	n	%
A-Con- Sub	Hydrothermal ageing and dynamic loading	Prefabricated	Subtractive	8	100	0	0	0	0
A-Con-PalaA			Pala A	0	0	8	100	0	0
A-Con-PalaB			Pala B	4	50	4	50	0	0
A-Con-SLA			SLA	0	0	0	0	7	100
A-Con-DLP			DLP	4	50	0	0	4	50
Con-Sub	No ageing	Prefabricated	Subtractive	5	62,5	0	0	3	37,5
Con-PalaA			Pala A	0	0	8	100	0	0
Con-PalaB			Pala B	2	25	0	0	6	75
Con-SLA			SLA	8	100	0	0	0	0
Con-DLP			DLP	1	12,5	0	0	7	87,5
A-CAD/CAM-Sub	Hydrothermal ageing and dynamic loading	CAD/CAM	Subtractive	6	75	0	0	2	25
A-CAD/CAM-SLA			SLA	0	0	8	100	0	0
A-CAD/CAM-DLP			DLP	0	0	8	100	0	0
CAD/CAM-Sub	No ageing	CAD/CAM	Subtractive	0	0	3	37,5	5	62,5
CAD/CAM-SLA			SLA	0	0	8	100	0	0
CAD/CAM-DLP			DLP	0	0	8	100	0	0

## Discussion

This in vitro study evaluated the bond strength and fatigue performance of denture materials fabricated with three different workflows: conventional, additive (SLA/DLP) and subtractive (milling). These were combined with either prefabricated or CAD/CAM, milled or printed, teeth. Statistically significant differences between the groups confirmed that the null hypothesis of equal performance across workflows had to be rejected. Group PalaA, which combined prefabricated Vita Vionic teeth with direct casting using PalaXpress resin, demonstrated the highest overall performance. This group achieved the highest bond strength before ageing (445.7 ± 49.2 N) and retained 92% of its strength after thermomechanical ageing (411.7 ± 45.7 N). Additionally, it demonstrated 100% survival in after hydrothermal and mechanical ageing. Furthermore, cohesive fractures were consistently observed before and after ageing. These results align with those of Choi et al.^9^, who found that teeth bonded to heat-cured materials exhibited the highest fracture toughness. However, they also noted a significant decrease in bond strength after thermocycling. This was explained by swelling and deterioration of the cross-linked matrix within the composite teeth, as well as leaching of components due to interface hydrolysis. It is known that these processes decrease the mechanical properties of PMMA^9,10^. The best synergy between heat-polymerized PMMA and compatible prefabricated teeth are achieved when denture teeth are embedded directly into freshly polymerizing PMMA using compression moulding with slow heat polymerization^11^. By contrast, alternative workflows usually require secondary bonding steps, which introduce more interface variability. In addition to mean fracture loads, standard deviations offer insights into the reproducibility of workflows. The conventional PalaA group exhibited a combination of high strength and low dispersion both prior to and following the ageing process (445.7 ± 49.2 N; 411.7 ± 45.7 N), suggesting a stable and predictable bonding interface. Conversely, a significant increase in variability was observed in multiple groups following the process of ageing. A particularly pronounced scatter was observed in A-CAD/CAM-SLA (312.8 ± 180.8 N), A-Con-Sub (84.1 ± 57.8 N), and A-Con-PalaB (190.9 ± 104.1 N), where standard deviations exceeded 50% of the mean. These findings suggest heterogeneous interfacial degradation rather than uniform strength reduction. Clinically, this dispersion implies a reduction in predictability, as individual specimens may perform substantially below the group mean despite acceptable average values. The degradation observed after thermomechanical cycling is consistent with polymer and composite engineering research demonstrating that environmental exposure progressively weakens bonded interfaces. Accelerated aging conditions involving temperature and moisture reduce fracture toughness and load-bearing capacity of adhesive joints and composite connections, confirming that interface deterioration rather than bulk material failure governs long-term mechanical performance^12,13^. The groups using additive manufacturing with SLA and DLP workflows demonstrated good performance, but with some limitations. Both the CAD/CAM-DLP and CAD/CAM-SLA groups exhibited 100% cohesive fracture patterns before and after ageing, suggesting robust bonding at the tooth–base interface. Following polymerization, a network of interwoven polymer chains is formed, which serve to join the denture base to the resin tooth. The strength of the bond is determined by the extent to which the monomer penetrates and by the strength of the subsequently formed interwoven polymer network^14^. The outcomes of the study were influenced by the type of bonding protocol that was utilized. CediTec primer contains Acetone as a solvent and HEMA. Acetone is a non-polymerizable organic solvent that has the potential to swell the surface, allowing the polymerizable small monomer HEMA to diffuse and thereby potentially increase the bond strength^15^. Research has indicated that the bond to the unreacted C = C double bonds and the surface roughness enhanced by sandblasting is of particular significance^15^. The conversion rate measured of the printed materials ranges from 74 to 85%, indicating a sufficient number of potential bonding partners. Materials with a lower degree of conversion, as may occur in SLA-printed components, retain unreacted double bonds that support chemical bonding^3,16^. This is likely one of the reasons why the SLA group maintained cohesive fracture patterns despite the absence of a bonding agent. While most groups used separate adhesive bonding for the purpose of joining the teeth and base, the SLA group relied entirely on its native 3D printable resin for the purpose of bonding. This material homogeneity may have facilitated chemical integration, which could explain the consistent cohesive fractures observed. A low conversion rate can be beneficial for primary bonding. The CAD/CAM-SLA group exhibited significant variability in bond strength after ageing (312.8 ± 180.8 N), suggesting potential susceptibility to long-term stability due to material degradation. The large standard deviation in this group indicates that ageing effects did not occur uniformly across specimens. While some samples maintained high fracture resistance, others showed substantial degradation, leading to a broad scatter of results. Such heterogeneity suggests localized interfacial weaknesses or inconsistencies in polymerization and wetting, rather than a uniform reduction in material strength. This may be due to inadequate wetting of the surface by the printing resin, which is more viscous compared to a acetone primer like CediTEC Adhesive. This may result in a potentially less interwoven network of polymer chains connecting the denture base to the acrylic tooth. Furthermore, hydrophilic monomers are frequently utilised as diluents in 3D printable resins, such as those employed by Formlabs. In combination with potential incomplete wetting and incomplete polymerization, due to also oxygen inhibition, they can result in water sorption penetrating the layers. This phenomenon is known to induce movement in the polymer chains, which can result in dimensional changes, debonding and lower mechanical properties^7,17,18^. As shown by Alrahlah et al.^19^, water uptake can range from 0.5% to over 2% by volume depending on the formulation, affecting structural integrity. Previous findings also showed that 3D-printed materials had stable fracture toughness after thermocycling, but inconsistencies were not uncommon^9^.

The CAD/CAM-DLP group exhibited a significant 57% decrease in bond strength following ageing, dropping from 317 ± 90 N to 181 ± 62 N. Although the post-ageing standard deviation was lower than in the SLA group, the scattering of bonding strength indicates that hydrothermal fatigue affected the interface to varying degrees. The combination of reduced mean bonding strength and sustained variability suggests progressive interfacial degradation rather than simple bulk material weakening. Furthermore, survival rates after hydrothermal ageing and dynamic loading decreased to 87.5%. These findings differ from the observations reported by Alharbi et al.^20^ who did not detect a significant reduction in bonding strength of 3D-printed denture systems after dynamic loading. However, it should be emphasized that their experimental protocol involved 250,000 loading cycles, whereas the present study applied 1.2 million cycles under simultaneous hydrothermal ageing. This higher number of cycles likely induced more pronounced interfacial fatigue, which may explain the divergent ageing effects observed. Therefore, while additive workflows may initially demonstrate stable bonding performance, prolonged hydrothermal fatigue appears to expose interface-dependent effects that become evident under extended cyclic loading conditions. These results suggest that the performance of additive workflows depends not only on chemical compatibility, but also on the material’s resistance to hydromechanical ageing. As demonstrated in the studies by Ferracane et al.^10^ and Alrahlah et al.^19^, plasticization effects can progressively reduce modulus and fracture resistance due to water uptake and matrix softening, particularly in formulations containing hydrophilic monomers such as UDMA, e.g. Voco dentbase at 50–100% and Formlabs at 25–35%, during hydrothermal fatigue.

The aged subtractive groups showed the lowest bonding strength. Subsequent to ageing, all subtractive groups exhibited predominantly adhesive failures, irrespective of whether prefabricated or CAD/CAM teeth had been used. Survival rates after hydrothermal and mechanical ageing ranged from 50 to 62.5%. These failures suggest that the tooth–base interface was the mechanical weak point. The relatively wide dispersion observed in the aged subtractive groups further supports the interpretation of unstable interfacial bonding, where minor differences in surface treatment or adhesive penetration may have resulted in markedly different fracture loads between specimens. Comparable behavior has been reported for additively manufactured polymer joints, in which cracks initiate at the bonding interface due to insufficient chemical interaction and stress concentration within the overlap area. Under cyclic loading, interfacial crack propagation becomes the dominant failure mechanism rather than fracture of the printed material itself^6^. Prpić et al.^21^ and Steinmassl et al.^22^ have reported that the high degree of conversion typical of milled PMMA materials results in fewer reactive sites being available for chemical bonding. The absence of residual monomer hinders the formation of interpenetrating polymer networks, which are necessary for stable adhesion^23,24^. Degree of conversion and crosslinking density clearly influence bonding performance. Highly polymerized CAD/CAM materials resist further copolymerization and provide little opportunity for chemical interaction at the interface.

Differences in thermal expansion between bonded materials generate stresses at the interface during temperature changes. Repeated thermal loading produces microcrack formation and interfacial damage accumulation, a mechanism commonly described in aged composite and adhesively bonded structures subjected to environmental exposure^12,13^. While coefficients of thermal expansion (CTE) were not measured in this study, previous research indicates that CTE mismatch is more likely to create fatigue-prone interfaces in highly cross-linked systems^8,16^. The vulnerability of subtractive specimens to cyclic thermal stress further supports this.

The outcomes of the study were influenced by the type of bonding protocol that was used. Whilst the majority of groups utilised separate adhesive bonding for the purpose of joining the teeth and base, the SLA group relied entirely on its native 3D printing resin for the purpose of bonding. It is interesting to note that, despite this apparent simplification, the SLA group outperformed several adhesive-reliant workflows, especially subtractive ones. This suggests that in cases where chemical compatibility and curing conditions are optimised, as they may be in a single-resin system, the omission of adhesives may not be disadvantageous. However, it should be noted that this also introduces variability depending on the printer, the material batch, and the quality of the post-curing process.

The analysis of fracture patterns lends further support to these conclusions. Cohesive fractures were observed in the PalaA, CAD/CAM-SLA, and CAD/CAM-DLP groups, indicating robust integration. In contrast, the utilisation of subtractive workflows has been observed to result in persistent adhesion failures, thereby underscoring the critical role of material chemistry and polymerisation behaviour in determining bond performance^9,16^.

A high bonding strength and a high survival rate reduce the risk of tooth detachment, thereby improving denture longevity and patient satisfaction while reducing long-term maintenance costs. The clinical relevance of bonding strength and material durability is particularly pronounced in geriatric prosthodontics. Older adults often have reduced manual dexterity, cognitive impairment or physical frailty, which makes repairing or replacing fractured prostheses more challenging^25^. While prefabricated teeth with conventional base resins remain a reliable choice, digital workflows can achieve similar performance if the bonding steps are fully optimised. The superior performance of conventional heat-polymerised resins with prefabricated teeth (Group PalaA) supports their continued use as a reliable option in denture fabrication. However, promising results from some additive manufacturing groups suggest that these newer technologies may offer viable alternatives, particularly in terms of digital workflow integration and customisation. The lower survival rates observed in subtractive manufacturing groups suggest that further optimisation of milling protocols or post-milling surface treatments is necessary to improve clinical performance. Additionally, the varied fracture patterns observed across the groups emphasise the importance of selecting the right materials and processing techniques to achieve durable tooth-denture base bonds.

Although this study provides valuable insights, its in vitro design cannot fully replicate the complex intraoral environment, including the presence of saliva, fluctuating pH values and multidirectional masticatory forces. Therefore, validation through clinical trials conducted under real-world conditions is essential to confirm these findings. Using standardised incisor geometry at the bonding interface may not capture the variability presented by different anatomical tooth shapes and manual laboratory procedures. Exploring a broader range of hydrothermal ageing and dynamic loading time points, and comparing samples that have only undergone hydrothermal ageing, could help to differentiate the observed effects more precisely.

Future research should investigate how varying surface treatments, bonding agents and post-processing methods influence the interface between the tooth and denture base. Post-curing protocols for additive manufacturing materials, including the impact of minor variations in post-curing steps or oxygen inhibition, should be investigated, as these may affect the degree of conversion and thereby alter bond strength. Comparative analyses of surface preparation methods across milled, printed and conventionally processed specimens would also be valuable. Understanding these interactions will be essential for refining digital denture fabrication protocols and ensuring their clinical reliability.

## Conclusion

Fabrication workflow significantly affects the long-term bonding durability between denture teeth and base materials under extended hydrothermal and mechanical fatigue. Conventional heat-polymerized PMMA with prefabricated teeth demonstrated the highest bonding strength. Additive CAD/CAM workflows showed promising initial performance but revealed material-dependent ageing effects after 1.2 million loading cycles. Subtractive workflows exhibited predominantly adhesive failures and reduced survival after ageing, indicating increased interfacial vulnerability. These findings underline the importance of optimizing bonding strategies in digital denture fabrication.

## Data Availability

The datasets generated during and/or analysed during the current study are available from the corresponding author on reasonable request.
